# Comprehensive analysis of pain genes in prognosis of kidney renal clear cell carcinoma and tumor immunotherapy: A comprehensive bioinformatic study

**DOI:** 10.1002/hsr2.1884

**Published:** 2024-02-13

**Authors:** Xiao‐Yu Zhuang, Ming Li, Da‐Ming Xu, Shu‐Bin Lin, Zheng‐Liang Yang, Teng‐Yu Xu, Jun Yin

**Affiliations:** ^1^ Department of Anesthesiology Second Affiliated Hospital of Shantou University Medical College Shantou People's Republic of China; ^2^ Department of Urology Second Affiliated Hospital of Shantou University Medical College Shantou People's Republic of China; ^3^ State Key Laboratory of Oncology in South China, Guangdong Provincial Clinical Research Center for Cancer Sun Yat‐sen University Cancer Center Guangzhou People's Republic of China; ^4^ Department of Urology Sun Yat‐sen University Cancer Center Guangzhou People's Republic of China; ^5^ Department of Clinical Laboratory Medicine Second Affiliated Hospital of Shantou University Medical College Shantou People's Republic of China

**Keywords:** immunomodulator, immunotherapy, kidney renal clear cell carcinoma, pain gene, prognosis

## Abstract

**Background:**

The effect of pain genes (NAV1, EHMT2, SP1, SLC6A4, COMT, OPRM1, OPRD1, CYP2D6, and CYP3A4) have not been reported previously in kidney renal clear cell carcinoma (KIRC) patients and thus we made a comprehensive analysis of pain genes in the prognosis of KIRC and tumor immunotherapy.

**Methods:**

In this study, TCGA, Kaplan–Meier plotter, Metascape, STRING, Human Protein Atlas, Single Cell Expression Atlas database, LinkedOmics, cBioPortal, MethSurv, CancerSEA, COSMIC database and R package (ggplot2, version 3.3.3) were used for comprehensive analysis of pain genes in KIRC. Pearson and Spearman correlation coefficients were for co‐expression analysis. Immunotherapy and TISIDB database were used for tumor Immunotherapy.

**Results:**

Representative pain genes (SP1, SLC6A4, COMT, OPRD1, CYP2D6, and CYP3A4) were statistically significant (*p* < 0.0001) in the prognosis of KIRC. Immunotherapy (anti‐PD‐1 therapy, anti‐PD‐L1 therapy, and anti‐CTLA4 therapy) and immunomodulator (immunoinhibitor, immunostimulator, and MHC molecule) in KIRC were significant associated with pain genes (SP1, SLC6A4, COMT, OPRD1, CYP2D6, and CYP3A4), which were the important addition to clinical decision making for patients.

**Conclusions:**

Our study uncovered a mechanism for the effect of pain genes on KIRC outcome via the modulation of associated co‐expression gene networks, gene variation, and tumor Immunotherapy.

## 1 INTRODUCTION

Kidney renal clear cell carcinoma (KIRC) is the most common subtype of renal cell carcinoma and one of the most common primary malignant tumors of the urinary system, also one of the top 10 common tumors in the world, ranking sixth among the top 10 tumors in male and ninth in female.^1^, ^2^ The other two common subtypes are papillary renal cell carcinoma and chromophobe renal cell carcinoma.^3^ Although renal cancer is a disease that can be detected early and treated surgically, one‐third of cases will progress to metastasis, which is associated with poor prognosis and poses great treatment challenges.^4^ Currently, kidney cancer is considered to be a disease closely related to genetic mutations. The genes that are mutated in kidney cancer are involved in different pathways and signaling to regulate various aspects, including angiogenesis,^5^ mitochondrial autophagy,^6^ DNA repair,^7^ intercellular junctions,^8^ extracellular matrix junctions,^9^ microtubule stability,^10^ mitotic spindle function,^11^ and cellular metabolism.^12^ At present, the following four key genes are considered as KIRC risk drivers, which are VHL, PBRM1, BAP1, and SETD2, respectively.^13^ Tumor‐specific neoantigens can provide more opportunities for cytotoxic T cells to interact with and destroy cancer cells to enhance the immune response.^14^ Notably, the treatment options for KIRC patients have increased over the past decade, with cancer immunotherapy becoming as an emerging and important field and a breakthrough to change adverse outcomes.^15^ Due to the different immunotherapy regimens, it is urgent to find biomarkers to identify tumor heterogeneity, so as to develop dosage regimens and predict drug efficacy. Therefore, it is meaningful to evaluated the correlation between biomarkers, tumor immunotherapy, and clinical outcomes for targeting or circumventing tumor heterogeneity.

Pain is the most desperate symptom of cancer, impairing life quality and shortening survival time.^16^ We have focused on pain management in cancer patients, yet the role of pain‐related metabolic responses in tumor pathophysiology remains a mystery. Of interest, opioid receptor has been reported to be expressed in sheer volume of human cancers, containing prostate cancer,^17^ lung cancer,^18^ and breast cancer.^19^ In retrospective studies of patients with prostate or lung cancer receiving opioids for pain, higher opioid needs were found to be associated with poorer cancer outcomes.^17^, ^18^ Pain genes are key mediators of the pain pathway.^20^ Due to the different mechanisms of pain, there are mainly four types of pain‐representative genes, including (a) ion channel pain genes: sodium ion channel (gene representative: Nav1), potassium ion channel (gene representative: EHMT2), calcium ion channel (gene representative: SP1); (b) neurotransmitter pain genes: 5‐hydroxytryptamine (gene representative: SLC6A4), catecholamine neurotransmitter (gene representative: COMT); (c) opioid receptor pain genes (gene representatives: OPRM1, OPRD1); (d) drug metabolism enzyme pain genes: cytochrome P4502D6 enzyme (gene representative: CYP2D6), cytochrome P4503A4 enzyme (gene representative: CYP3A4).

As the few studies on the pain molecular basis of KIRC, we believe that pain genes (NAV1, EHMT2, SP1, SLC6A4, COMT, OPRM1, OPRD1, CYP2D6, and CYP3A4) have not been reported previously in patient with KIRC. These findings provide a new breakthrough for prognosis and treatment in KIRC.

## 2 MATERIALS AND METHODS

TIMER database (http://timer.cistrome.org) was applied for the pan‐cancer analysis of different pain genes. TCGA database (https://portal.gdc.cancer.gov) was used for expression level analysis and clinical characteristics analysis of different pain genes in KIRC patients via R package (ggplot2, version 3.3.3). Kaplan–Meier plotter database (http://www.kmplot.com) was performed to make a survival significance analysis of different pain genes. Metascape database(https://metascape.org) was used to make the pathway and process enrichment analysis and quality control and association analysis of pain genes. STRING database (https://version11.string-db.org) was applied for the molecule interaction analysis of pain genes. Human Protein Atlas database (https://www.proteinatlas.org) was used for protein expression level and subcellular location expression level analysis. Single Cell Expression Atlas database (https://www.ebi.ac.uk/gxa/sc/home) was allowed to perform a spatiotemporal immune zonation analysis. LinkedOmics database (https://www.linkedomics.org/login.php) was used for positively correlated significant gene expression and negatively correlated significant gene expression analysis of different pain genes in KIRC patients. cBioPortal database (https://www.cbioportal.org) was applied for DNA methylation analysis of different pain genes. MethSurv database (https://biit.cs.ut.ee/methsurv) was used for Heatmap analysis of DNA methylation. CancerSEA database (http://biocc.hrbmu.edu.cn/CancerSEA/home.jsp) was performed to make an associated cancer functional states analysis of different pain genes. COSMIC database (https://cancer.sanger.ac.uk/cosmic) was used for gene mutation distribution analysis of different pain genes. The Immunotherapy database (https://www.rocplot.org/immune) was used for immunotherapy analysis, containing anti‐PD‐1 therapy, anti‐PD‐L1 therapy, and anti‐CTLA4 therapy of different pain genes TISIDB database (http://cis.hku.hk/TISIDB/index.php) was applied for immunomodulator analysis, containing immunoinhibitor, immunostimulator and MHC molecule of different pain genes.

## 3 RESULTS

### 3.1 Pan cancer analysis of pain genes (NAV1, EHMT2, SP1, SLC6A4, COMT, OPRM1, OPRD1, CYP2D6, and CYP3A4) in KIRC patients

**Figure 1:**
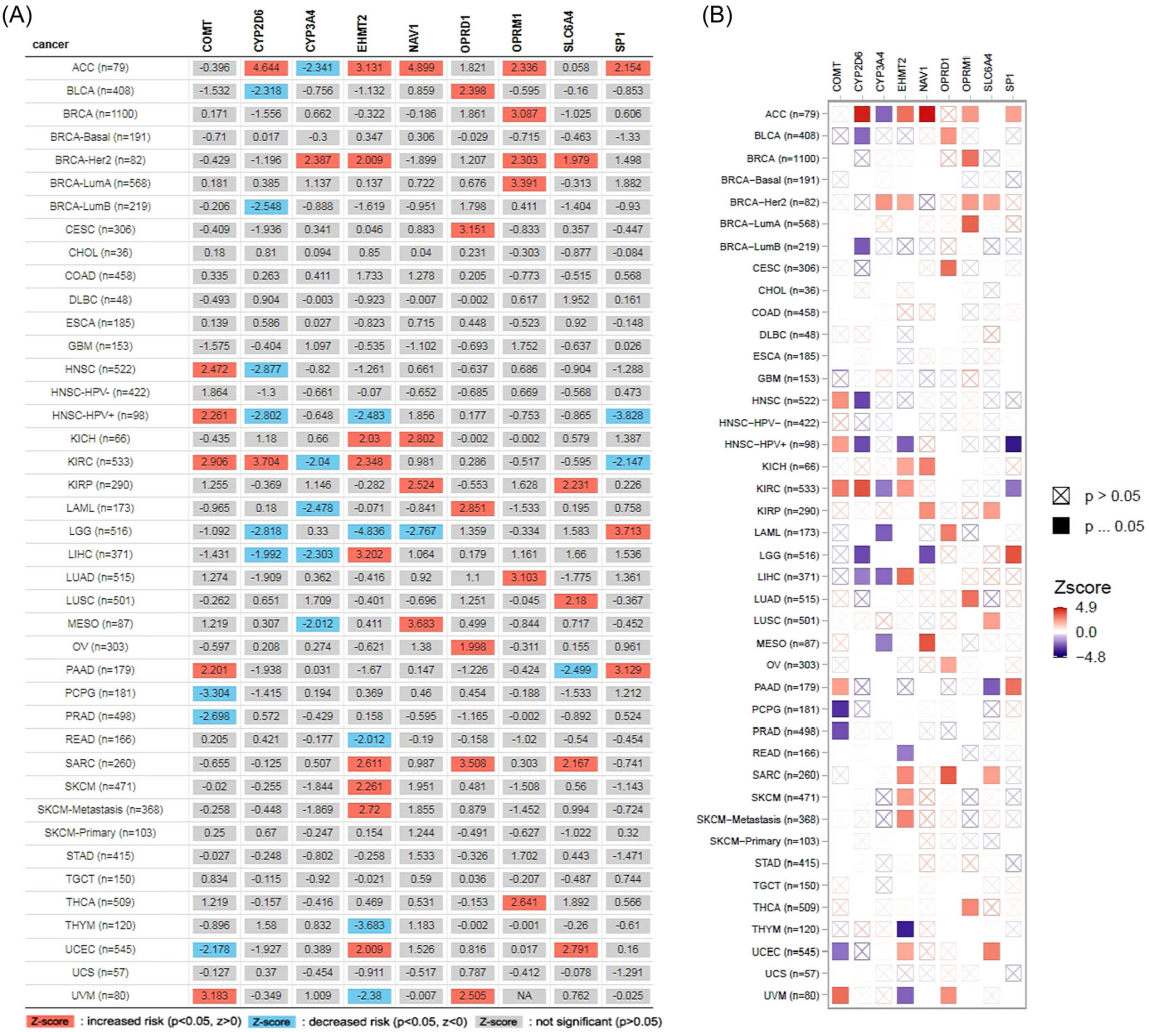
Heatmap of pain genes expression in various tumors. (A) Different prognostic significance in pain genes. (B) Heatmap of pan cancer analysis.

TIMER database was used for pan‐cancer analysis of different pain genes (Nav1, EHMT2, SP1, SLC6A4, COMT, OPRM1, OPRD1, CYP2D6, and CYP3A4) (Table 1). The expression of pain genes was statistically significant in a variety of tumors with different prognostic significance (Figure 1).

**Table 1:** Summary of target pain genes.

Genes	Description	Chromosomal Location
NAV1	Neuron navigator 1	1q32.3
EHMT2	Euchromatic histone‐lysine N‐methyltransferase 2	6p21.3
SP1	Sp1 transcription factor	12q13.1
SLC6A4	Solute carrier family 6 member 4	17q11.2
COMT	Catechol‐*O*‐methyltransferase	22q11.21
OPRM1	Opioid receptor, mu 1	6q24−q25
OPRD1	Opioid receptor, delta 1	1p36.1−p34.3
CYP2D6	Cytochrome P450, family 2, subfamily D, polypeptide 6	22q13.1
CYP3A4	Cytochrome P450, family 3, subfamily A, polypeptide 4	7q21.1

### 3.2 Different pain genes (NAV1, EHMT2, SP1, SLC6A4, COMT, OPRM1, OPRD1, CYP2D6, and CYP3A4) expression level, survival significance, and predicted functional pathways in KIRC patients

**Figure 2:**
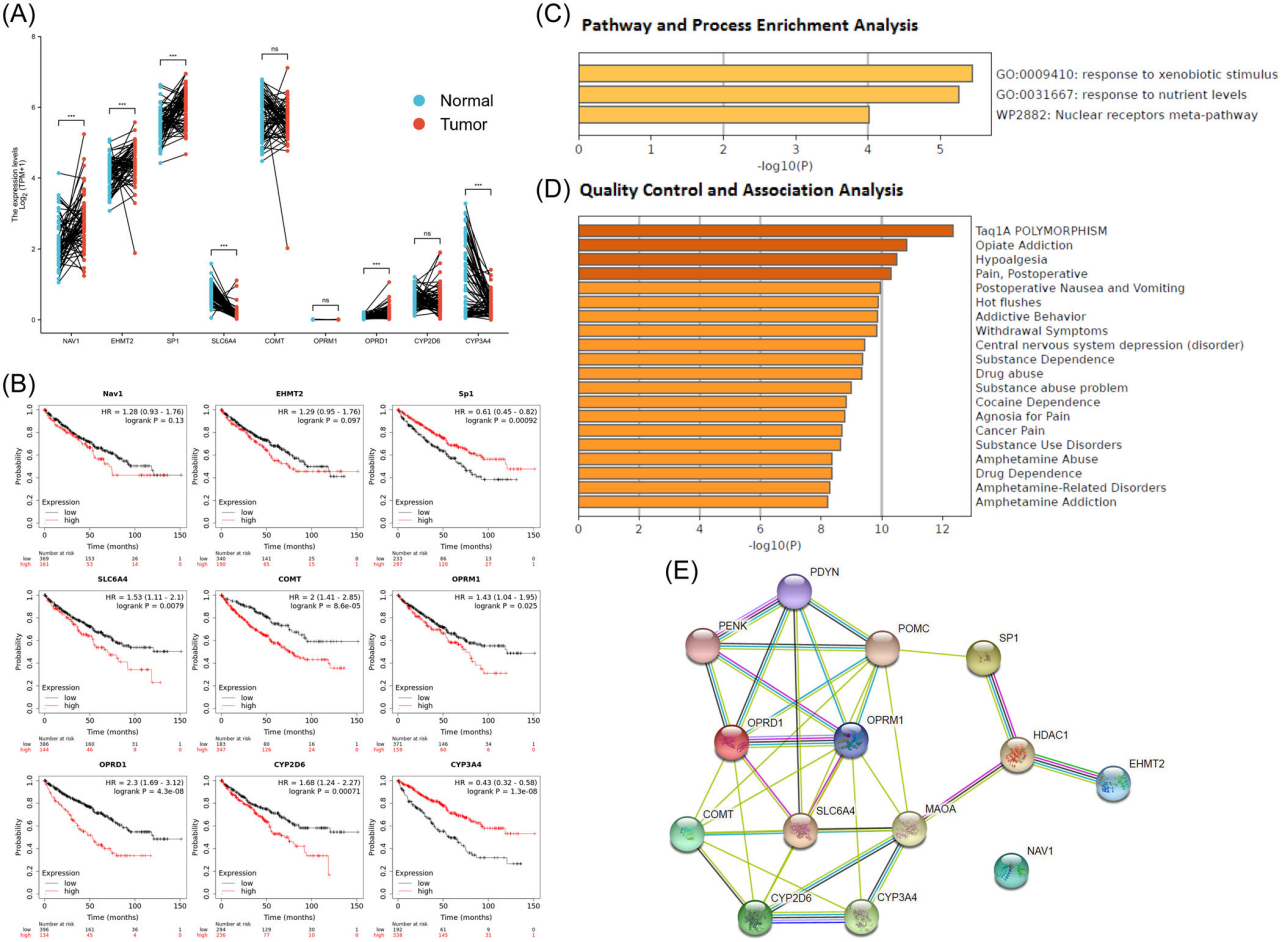
Different pain genes expression level, survival significance, and predicted functional pathways in kidney renal clear cell carcinoma. (A) The expression of pain genes in KIRC. (B) The prognosis of pain genes in KIRC. (C) Pathway and process enrichment analysis of pain genes. (D) Quality control and association analysis of pain genes. (E) Protein‐protein Interaction enrichment analysis of pain genes.

TCGA database was used to analyze expression levels of different pain genes (Nav1, EHMT2, SP1, SLC6A4, COMT, OPRM1, OPRD1, CYP2D6, and CYP3A4) in KIRC patients using R package (ggplot2, version 3.3.3) (Figure 2A). Kaplan–Meier plotter database was used for survival significance analysis of different pain genes (NAV1, EHMT2, SP1, SLC6A4, COMT, OPRM1, OPRD1, CYP2D6, and CYP3A4) in KIRC patients. SP1 (HR = 0.61 and *p* = 0.00092) and CYP3A4 (HR = 0.43 and *p* = 1.3e−8) were as favorable survival biomarker for KIRC patients. SLC6A4 (HR = 1.53 and *p* = 0.0079), COMT (HR = 2 and *p* = 8.6e−5), OPRM1 (HR = 1.43 and *p* = 0.025), OPRD1 (HR = 2.3 and *p* = 4.3e−8) and CYP2D6 (HR = 1.68 and *p* = 0.00071) were as unfavorable survival biomarker for KIRC patients. In addition, NAV1 (HR = 1.28 and *p* = 0.13), EHMT2 (HR = 1.29 and *p* = 0.097) were not significantly associated with survival in KIRC patients (Figure 2B). Metascape database was used to make the pathway and process enrichment analysis and quality control and association analysis of pain genes. Pathway and process enrichment analysis showed the top 3 representative enriched clusters consisted of GO:0009410 (response to xenobiotic stimulus), GO:0031667 (response to nutrient levels) and WP2882 (nuclear receptors meta‐pathway) (Figure 2C). Quality control and association analysis showed that top 5 enriched clusters in DisGeNET were C4694057 (Taq1A POLYMORPHISM), C0524662 (opiate addiction), C0085625 (hypoalgesia), C0030201 (pain, postoperative) and C0520909 (postoperative nausea and vomiting) (Figure 2D). STRING database was used for protein–protein interaction enrichment analysis of different pain genes (Nav1, EHMT2, SP1, SLC6A4, COMT, OPRM1, OPRD1, CYP2D6, and CYP3A4) in KIRC patients (Figure 2E). Genes with survival *p* < 0.001 were included in our follow‐up study.

### 3.3 Clinical characteristics analysis of pain genes (SP1, SLC6A4, COMT, OPRD1, CYP2D6, and CYP3A4) in KIRC patients

**Figure 3:**
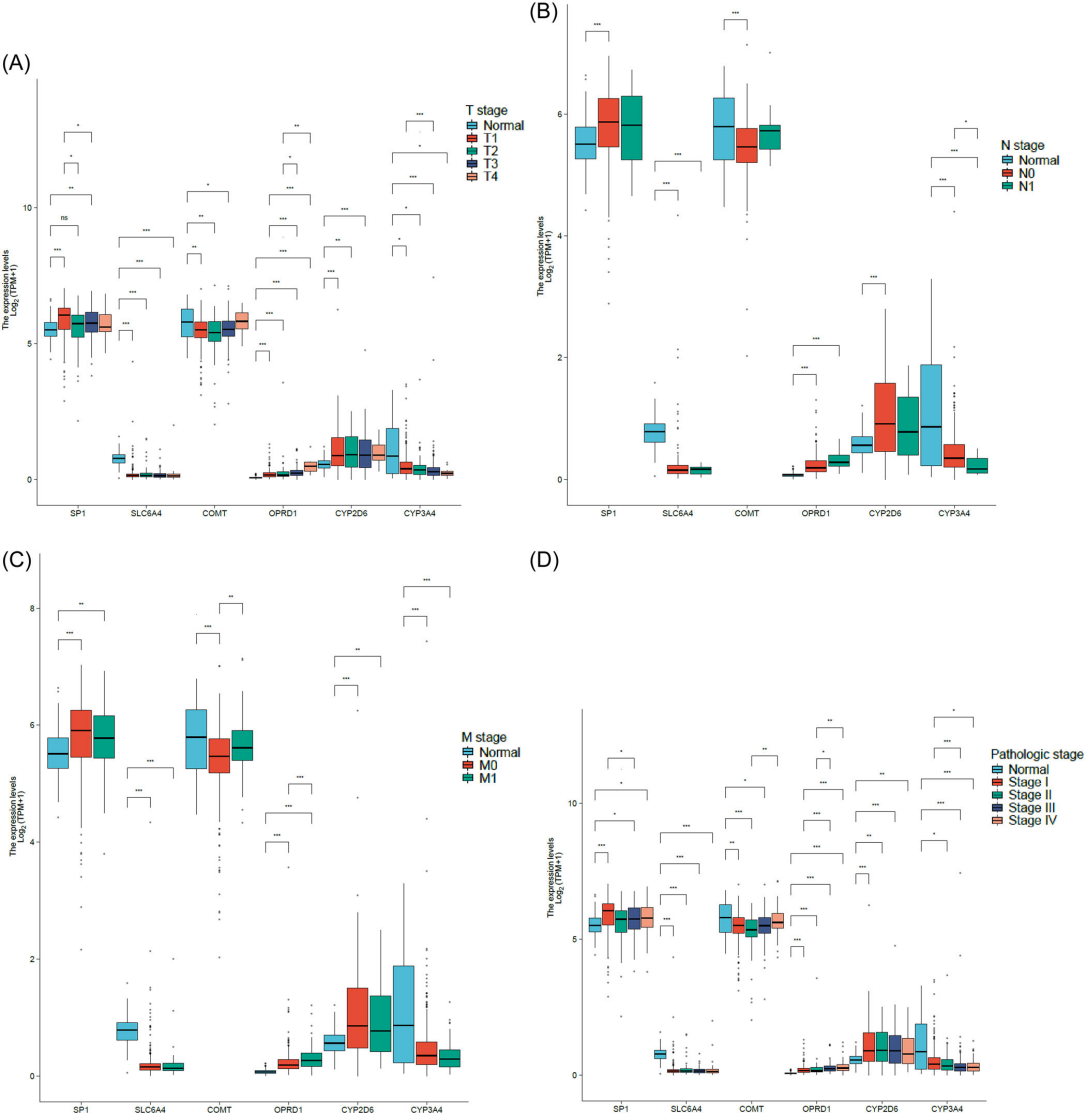
Associated clinical characteristics of pain genes in kidney renal clear cell carcinoma. (A) T stage of pain genes in KIRC. (B) N stage of pain genes in KIRC. (C) M stage of pain genes. (D) Pathologic stage of pain genes.

TCGA database was used to analyze clinical characteristics (T stage, N stage, M stage, and pathologic stage) of different pain genes (SP1, SLC6A4, COMT, OPRD1, CYP2D6, and CYP3A4) in KIRC patients via R package (ggplot2, version 3.3.3) (Figure 3).

### 3.4 Protein expression level, subcellular location expression level, and spatiotemporal immune zonation of pain genes (SP1, SLC6A4, COMT, OPRD1, CYP2D6, and CYP3A4)

**Figure 4:**
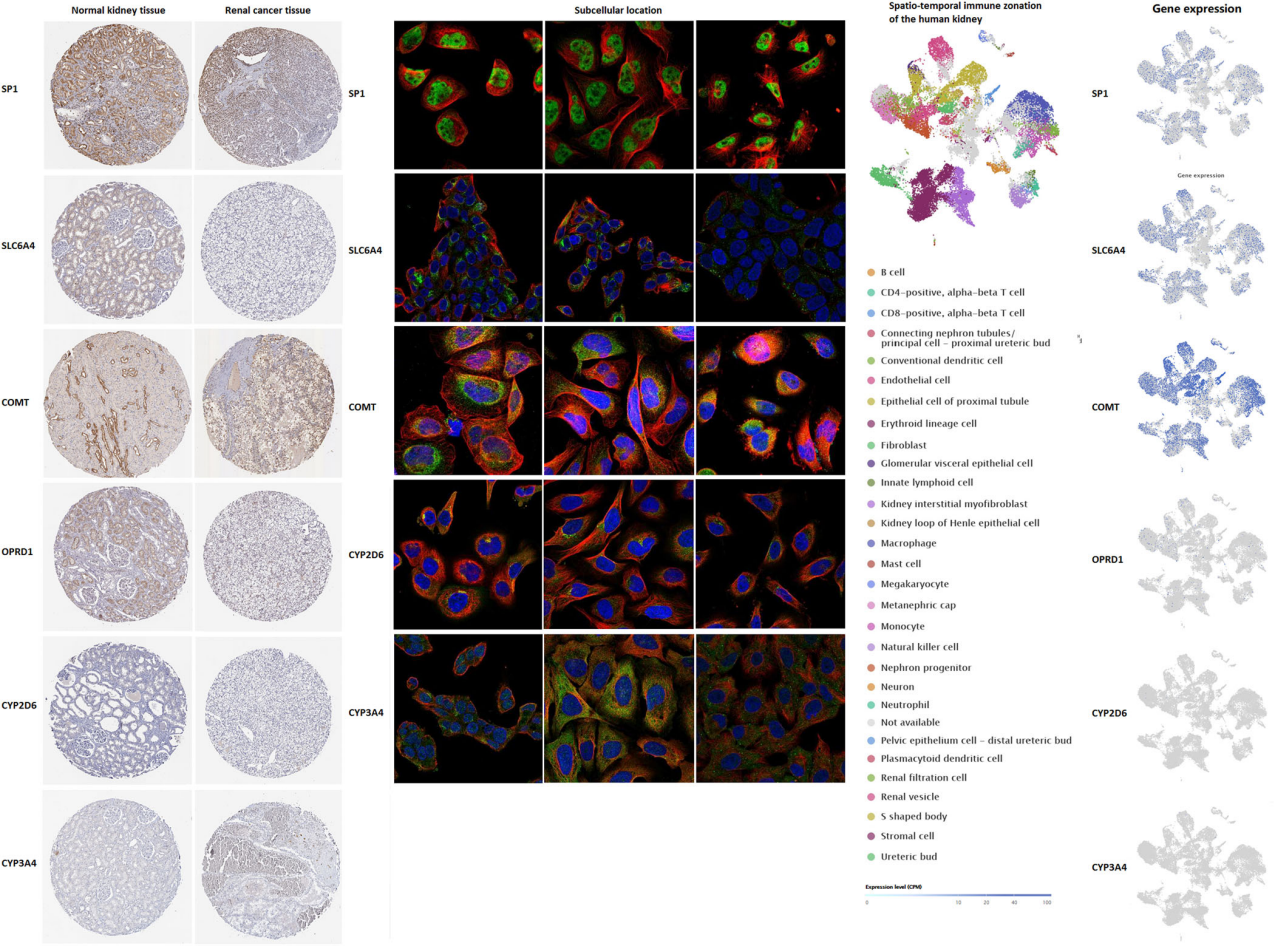
Protein expression level, subcellular location expression level, and spatiotemporal immune zonation of pain genes.

Human Protein Atlas database was used for protein expression level and subcellular location expression level analysis, and Single Cell Expression Atlas database was used for spatiotemporal immune zonation analysis (Figure 4). The protein expression of SP1 in renal tumor tissues was higher than that in normal kidney tissues. However, the protein expression of SLC6A4, COMT, and OPRD1 in renal tumor tissues was lower than that in normal kidney tissues. Additionally, CYP2D6 and CYP3A4 protein expression could not be detected in either renal tumor tissues or normal kidney tissues. The main subcellular location expression of SP1 was nucleoplasm. The main subcellular location expression of SLC6A4 were Golgi apparatus and vesicles. The main subcellular location expression of COMT was vesicles. The main subcellular location expression of CYP2D6 was in Golgi apparatus. The main subcellular location of CYP3A4 was cytosol.

### 3.5 Gene co‐expression analysis of pain genes (SP1, SLC6A4, COMT, OPRD1, CYP2D6, and CYP3A4)

**Figure 5:**
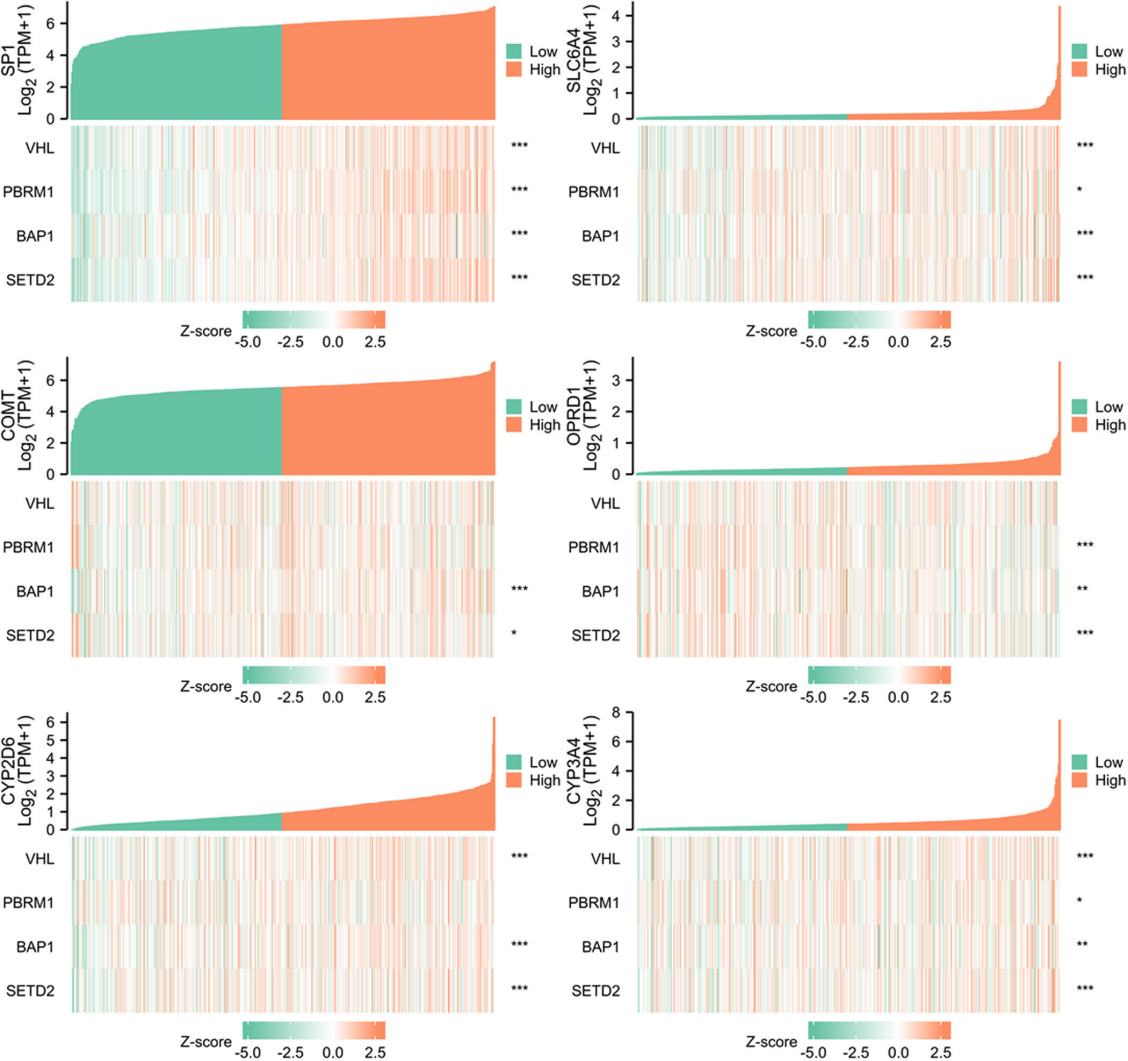
Association between kidney renal clear cell carcinoma risk driver genes and pain genes.

**Figure 6:**
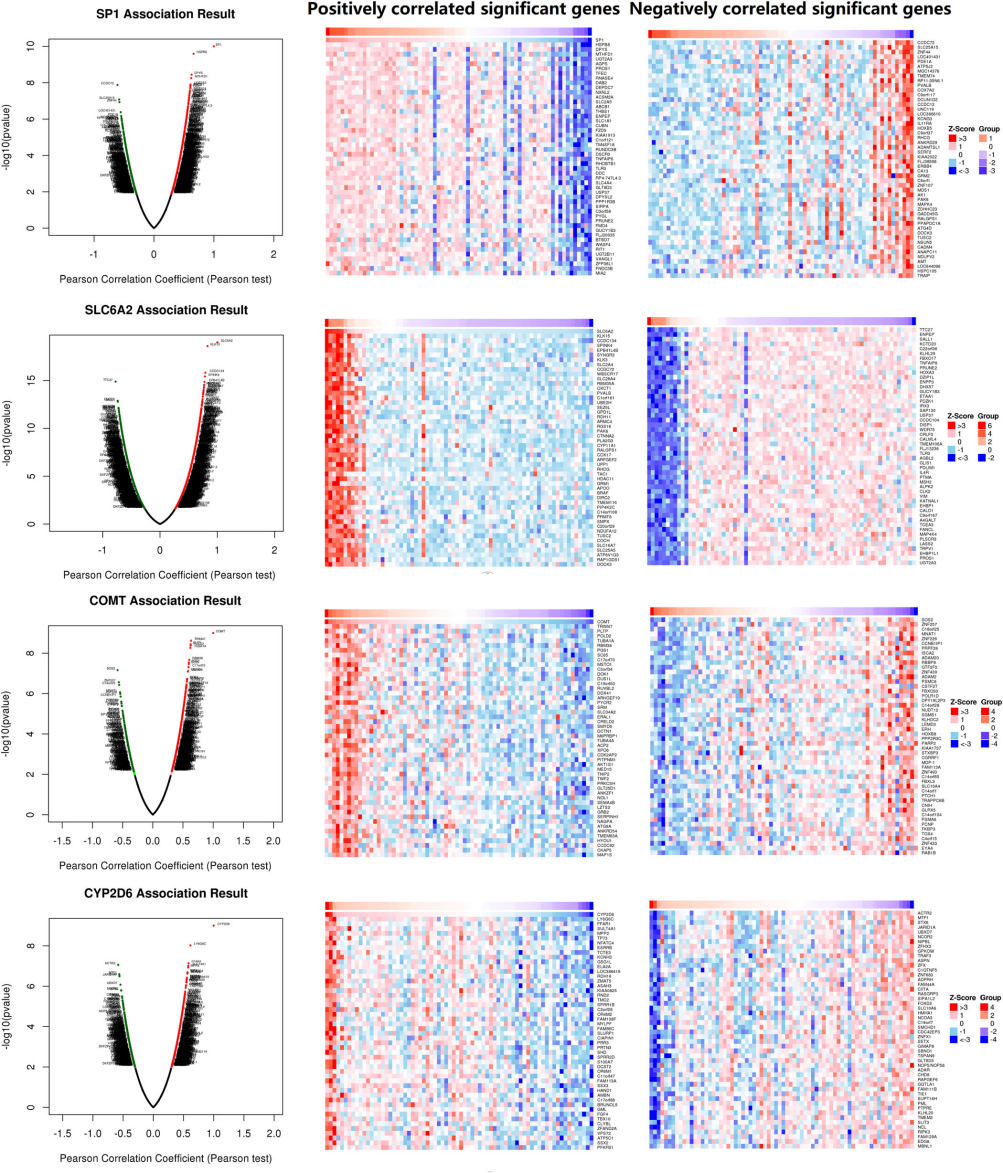
Heatmap of gene co‐expression of pain genes in kidney renal clear cell carcinoma.

TCGA database was used for KIRC risk driver genes (VHL, PBRM1, BAP1, and SETD2) co‐expression analysis of different pain genes via R package (ggplot2, version 3.3.3), which revealed the interaction between pain genes and KIRC risk driver genes (VHL, PBRM1, BAP1 and SETD2) (Figure 5, Table 2). LinkedOmics database was used for positively correlated significant gene expression and negatively correlated significant gene expression analysis of different pain genes in KIRC patients (Figure 6).

**Table 2:** Heatmap of co‐expression between pain genes and KIRC risk driver genes.

Pain gene	Risk gene	*r* (Pearson)	*p* Value	*r* (Spearman)	*p* Value
SP1	VHL	0.576	<0.001	0.546	<0.001
SP1	PBRM1	0.662	<0.001	0.640	<0.001
SP1	BAP1	0.404	<0.001	0.448	<0.001
SP1	SETD2	0.700	<0.001	0.670	<0.001
SLC6A4	VHL	0.198	<0.001	0.289	<0.001
SLC6A4	PBRM1	0.090	0.036	0.098	0.022
SLC6A4	BAP1	0.164	<0.001	0.224	<0.001
SLC6A4	SETD2	0.138	0.001	0.201	<0.001
COMT	VHL	−0.000	0.992	0.019	0.666
COMT	PBRM1	0.027	0.537	0.084	0.050
COMT	BAP1	0.368	<0.001	0.274	<0.001
COMT	SETD2	0.073	0.091	0.109	0.011
OPRD1	VHL	0.011	0.800	−0.001	0.980
OPRD1	PBRM1	−0.097	0.024	−0.164	<0.001
OPRD1	BAP1	−0.092	0.033	−0.141	0.001
OPRD1	SETD2	−0.143	<0.001	−0.174	<0.001
CYP2D6	VHL	0.252	<0.001	0.317	<0.001
CYP2D6	PBRM1	0.028	0.523	0.033	0.446
CYP2D6	BAP1	0.218	<0.001	0.233	<0.001
CYP2D6	SETD2	0.158	<0.001	0.172	<0.001
CYP3A4	VHL	0.112	0.009	0.198	<0.001
CYP3A4	PBRM1	0.045	0.295	0.092	0.032
CYP3A4	BAP1	0.070	0.105	0.112	0.009
CYP3A4	SETD2	0.120	0.005	0.184	<0.001

Abbreviation: KIRC, kidney renal clear cell carcinoma.

### 3.6 DNA methylation analysis of pain genes (SP1, SLC6A4, COMT, CYP2D6 and CYP3A4)

**Figure 7:**
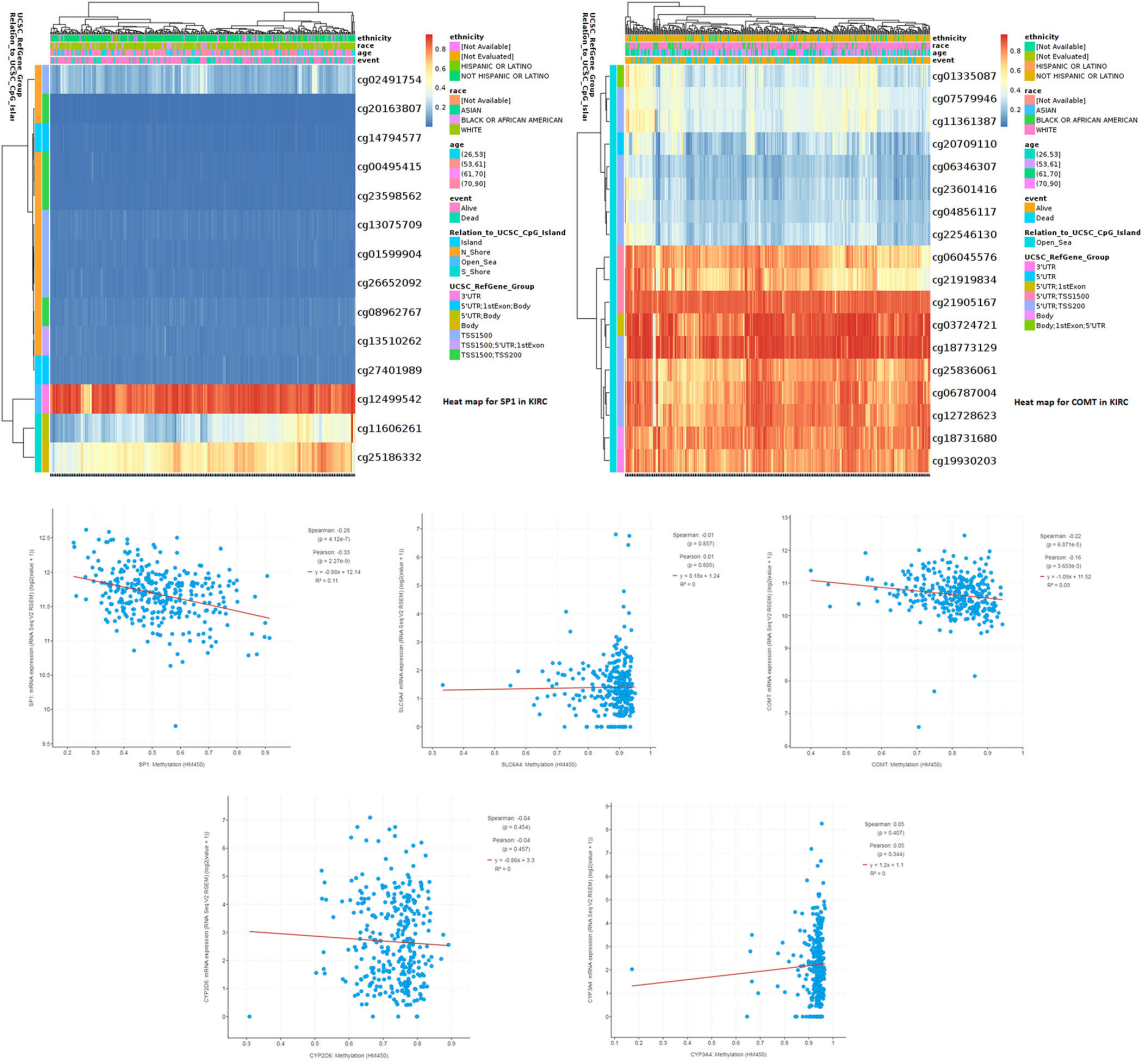
DNA methylation of pain genes in kidney renal clear cell carcinoma.

cBioPortal database was used for DNA methylation analysis of different pain genes. MethSurv database was used for Heatmap analysis of SP1 (*p* = 4.12e−7) and COMT(*p* = 6.87e−5) DNA methylation to reveal methylation sites (Figure 7).

### 3.7 Gene mutation distribution analysis of pain genes (SP1, SLC6A4, COMT, OPRD1, CYP2D6, and CYP3A4)

**Figure 8:**
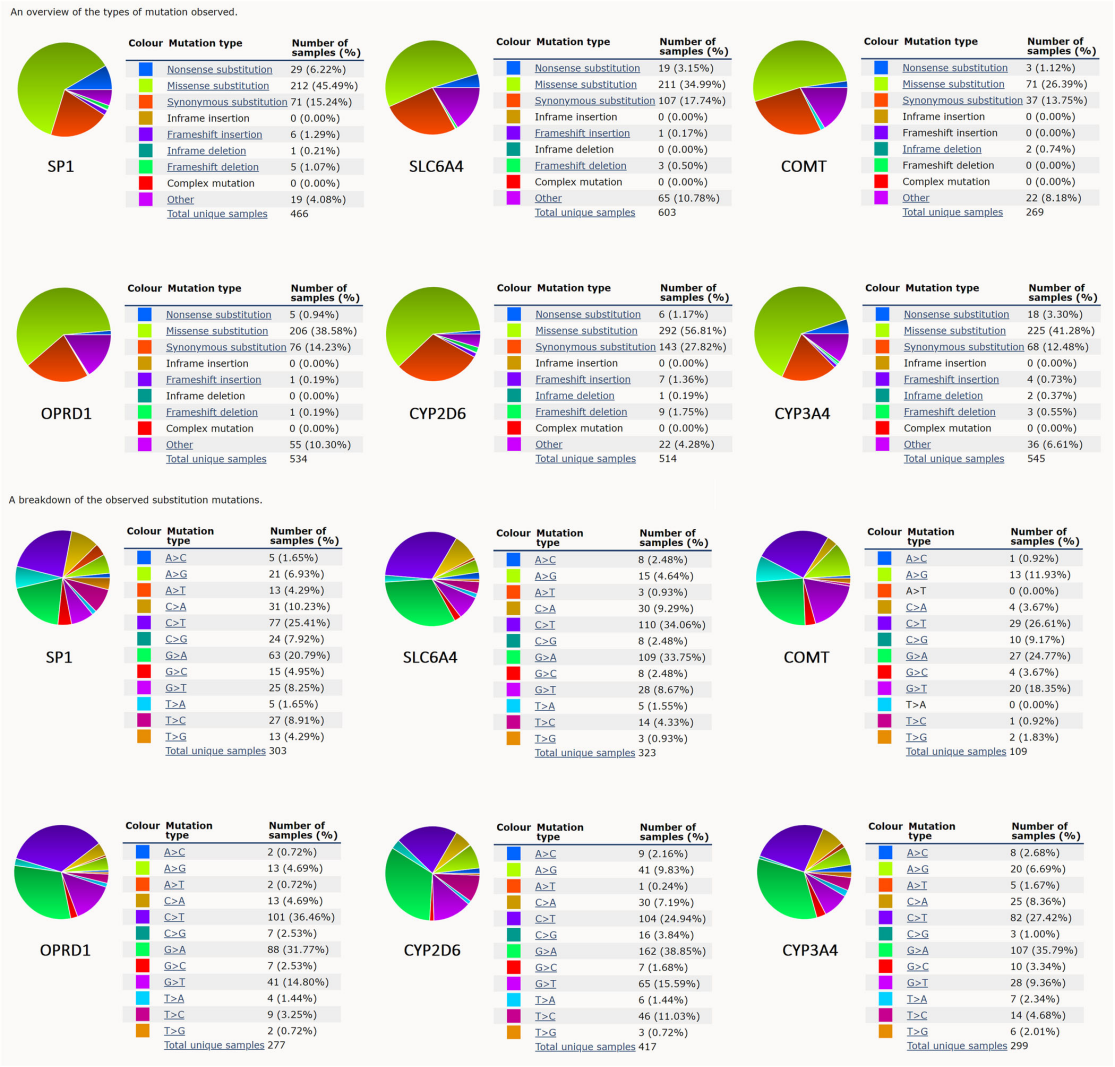
Gene mutation distribution of pain genes.

COSMIC database was used for gene mutation distribution analysis of different pain genes, including an overview of the mutation types and the breakdown of the substitution mutations (Figure 8).

### 3.8 Associated cancer functional states analysis of pain genes (SP1, SLC6A4, COMT, OPRD1, and CYP3A4)

**Figure 9:**
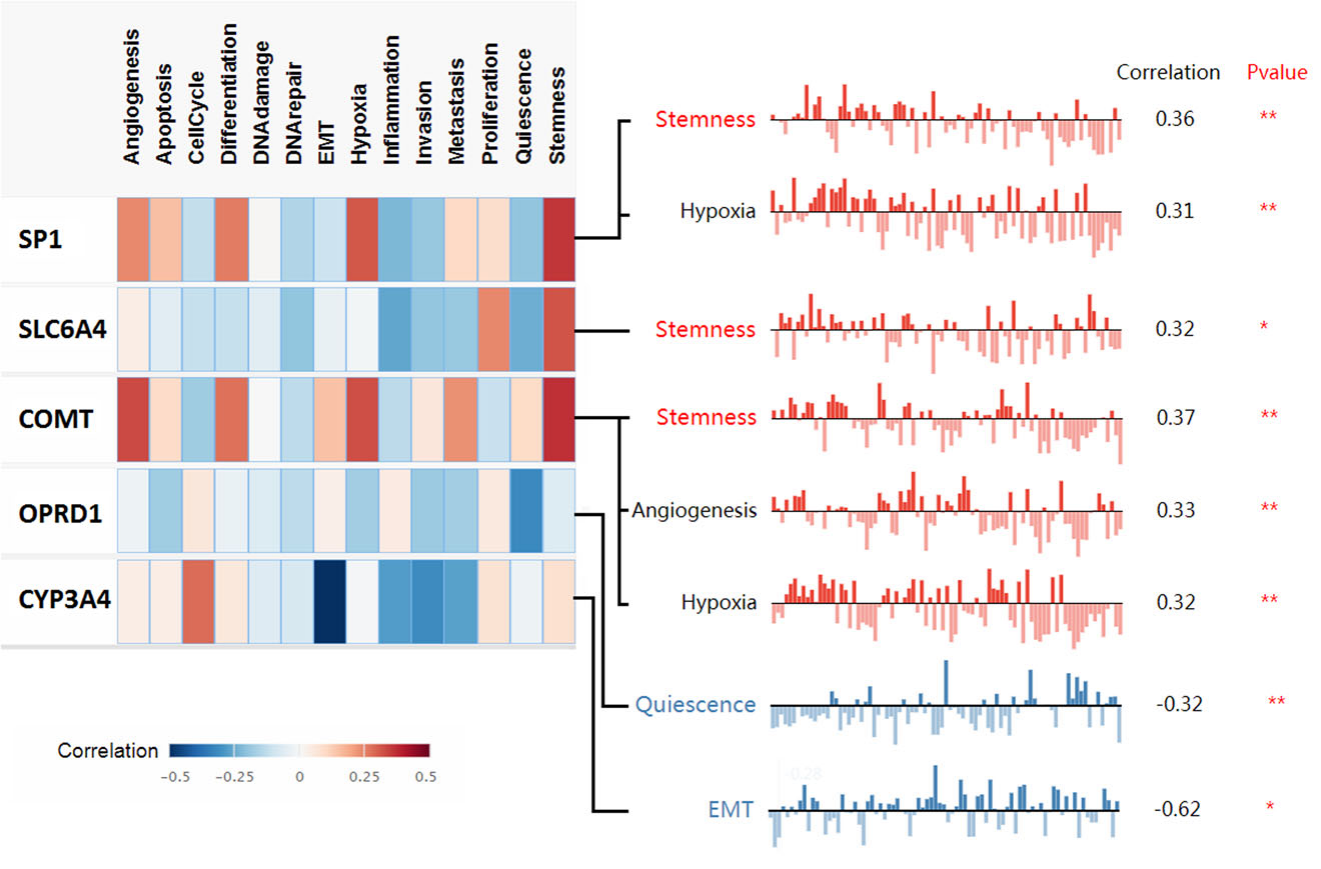
Associated cancer functional states of pain genes in kidney renal clear cell carcinoma.

CancerSEA database was used to analyze the associated cancer functional states of different pain genes (Figure 9). The top three cancer functional states of different pain genes in KIRC were stemness, hypoxia, and angiogenesis.

### 3.9 Immunotherapy analysis (anti‐PD‐1 therapy, anti‐PD‐L1 therapy, and anti‐CTLA4 therapy) and immunomodulator analysis (immunoinhibitor, immunostimulator, and MHC molecule) of pain genes (SP1, SLC6A4, COMT, OPRD1, CYP2D6, and CYP3A4)

**Figure 10:**
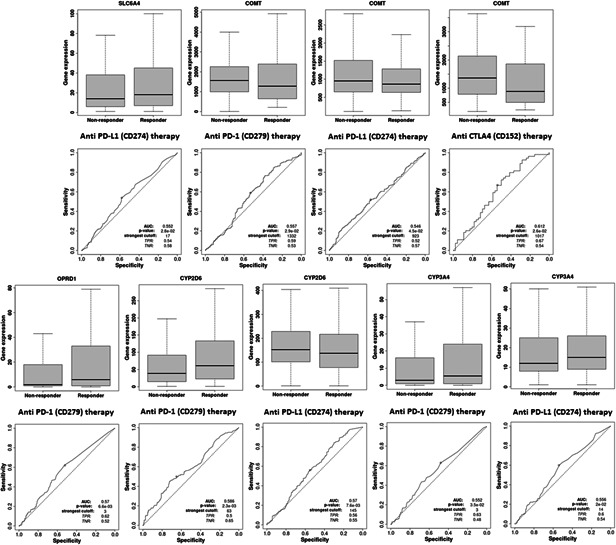
Association between immunotherapy (anti‐PD‐1 therapy, anti‐PD‐L1 therapy, and anti‐CTLA4 therapy) and pain genes.

**Figure 11:**
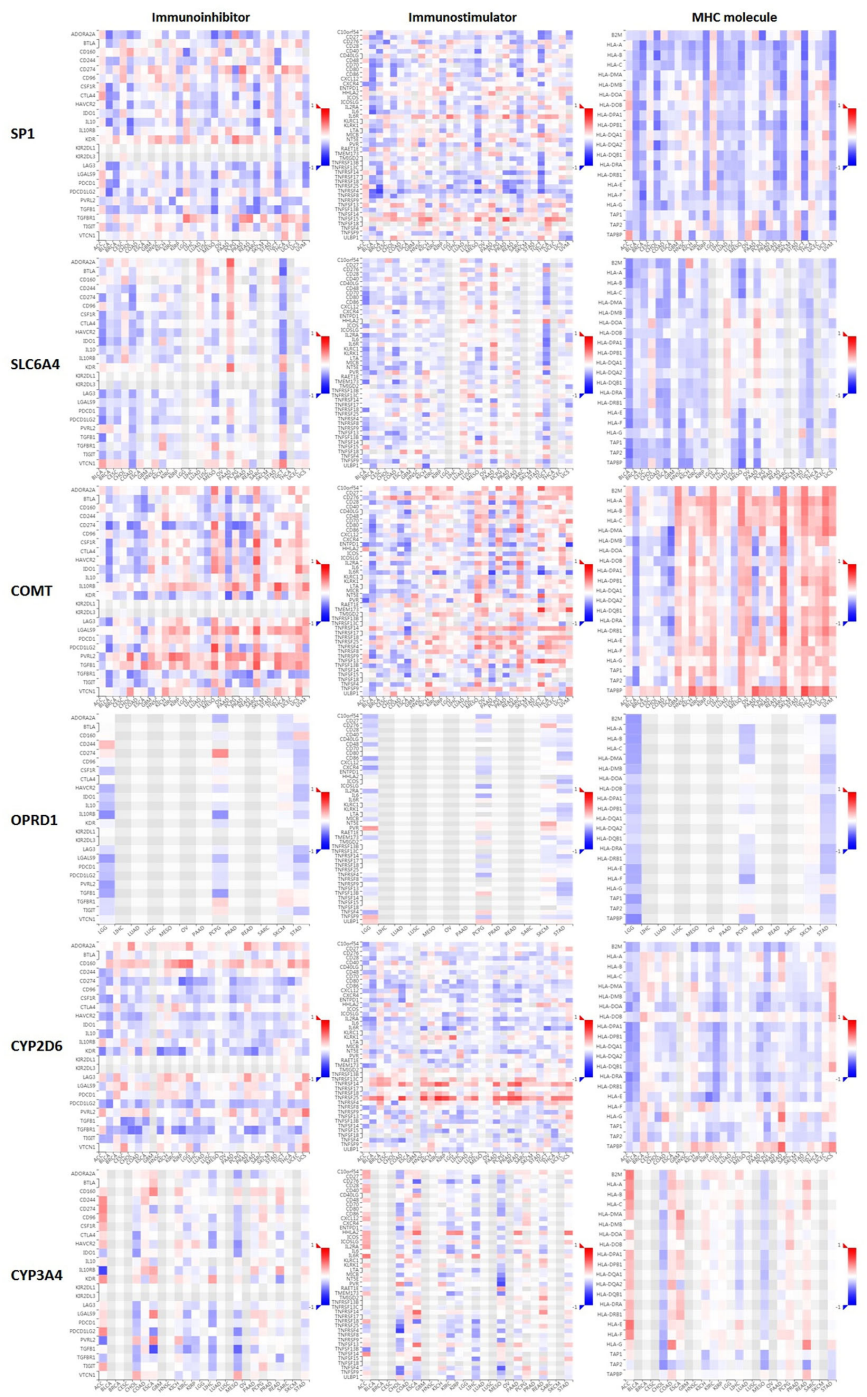
Association between immunomodulator (immunoinhibitor, immunostimulatory, and MHC molecule) and pain genes.

Immunotherapy database was used for immunotherapy analysis (anti‐PD‐1 therapy, anti‐PD‐L1 therapy, and anti‐CTLA4 therapy) of different pain genes (Figure 10). COMT (AUC = 0.557, ROC *p* = 2.92−02), OPRD1 (AUC = 0.57, ROC *p* = 6.6e−03), CYP2D6 (AUC = 0.586, ROC *p* = 2.3e−03) and CYP3A4 (AUC = 0.552, ROC *p* = 3.5e−02) were significant sensitivity to anti‐PD‐1 therapy. SLC6A4 (AUC = 0.552, ROC *p* = 2.8e−02), COMT (AUC = 0.546, ROC *p* = 4.5e−02), CYP2D6 (AUC = 0.57, ROC *p* = 7.6e−03) and CYP3A4 (AUC = 0.556, ROC *p* = 2e−02) were significant sensitivity to anti‐PD‐L1 therapy. COMT (AUC = 0.612, ROC *p* = 2.6e−02) were significant sensitivity to anti‐CTLA4 therapy. The immunomodulator analysis (immunoinhibitor, immunostimulator, and MHC molecule) of different pain genes also has been showed based on TISIDB database (Figure 11).

## 4 DISCUSSION

Aberrant gene expression is an important driver of KIRC,^13^ and is related to various pathophysiological processes of tumor.^5^, ^6^, ^7^, ^8^, ^9^, ^10^, ^11^, ^12^ Individualized tumor immunotherapy based on genes can provide the best therapeutic effect with minimal side effects for cancer patients. Of note, a preclinical study suggested that high expression of opioid receptor can activate the receptors that play critical roles in cancer progression or metastasis, including epidermal growth factor receptor, vascular endothelial growth factor tyrosine kinases receptor, platelet‐derived growth factor receptor, and mitogen‐activated protein kinase receptor.^21^, ^22^ Based on this, we first explored the expression levels of representative pain genes (NAV1, EHMT2, SP1, SLC6A4, COMT, OPRM1, OPRD1, CYP2D6, and CYP3A4) in KIRC, their prognostic significance, their roles in cancer functional states, their DNA methylation and their relevance to tumor immunotherapy (anti‐PD‐1 therapy, anti‐PD‐L1 therapy, and anti‐CTLA4 therapy) and immunomodulator (Iimmunoinhibitor, immunostimulator, and MHC molecule), which added crucial new members to the genomics of KIRC and provided the new references for clinical immune personalized therapy.

Our study revealed that the expression levels of NAV1, EHMT2, SP1, SLC6A4, OPRD1, and CYP3A4 between the normal and tumor were statistically significant. Representative pain genes (SP1, SLC6A4, COMT, OPRD1, CYP2D6, and CYP3A4) were statistically significant (*p* < 0.0001) in the prognosis of KIRC, and have been listed as the key research objects in follow study. Interestingly, after further study, we found that the expression differences of pain genes (SP1, SLC6A4, COMT, OPRD1, CYP2D6, and CYP3A4) were statistically significant in T stage, N stage, M stage, and pathological stage of KIRC. In addition, the gene co‐expression and DNA methylation sites were revealed. Besides, cancer functional states analysis of pain genes indicated that top 3 cancer functional states in KIRC were stemness, hypoxia, and angiogenesis, which suggested a possible pathological process in which the pain genes may be involved in renal cancer. Moreover, immunotherapy (anti‐PD‐1 therapy, anti‐PD‐L1 therapy, and anti‐CTLA4 therapy) and immunomodulator (immunoinhibitor, immunostimulator, and MHC molecule) in KIRC were significant associated with pain genes (SP1, SLC6A4, COMT, OPRD1, CYP2D6, and CYP3A4), which were the important addition to clinical decision making for patients.

### 4.1 Ion channel pain genes in KIRC

SP1, Sp1 transcription factor, a representative of Ca ion channel pain genes. Liu et al. found that Inhibition of SP1 ubiquitination in lncRNA SNHG12‐SP1‐CDCA3 axis can enhance the proliferation and invasion of KIRC and induce drug resistance to sunitinib.^23^ Overexpression of SP1 was also observed in other tumors and was associated with poor prognosis, which was consistent with our findings in KIRC. Pan et al. demonstrated that overexpression of SP1 can promote drug resistance to gefitinib and Osimertinib in non‐small cell lung cancer via the activation of EGFR signaling.^24^ SP1 can up‐regulate the expression level of lncRNA LINC00659 to promote the tumor progression of gastric cancer through mir‐370‐AQP3 axis, which was associated with lymph node metastasis and poor prognosis.^25^ Various evidence suggested that SP1 can activate the expression of oncogenes and suppress the expression of tumor suppressor genes, as well as participate in some basic cell functions, including cellular proliferation, differentiation and apoptosis, ultimately leading to poor prognosis.^26^ Therefore, SP1 was an essential prognostic biomarker in KIRC.

### 4.2 Neurotransmitter pain genes in KIRC

SLC6A4, solute carrier family 6 member 4, a representative of 5‐hydroxytryptamine pain genes. It has been reported that variants in the SLC6A4 gene were associated with unfavorable prognosis in patients with colorectal cancer.^27^ 5‐Hydroxytryptamine may enhance tumorigenicity and increase the risk of distant metastasis in non‐small cell lung cancer patients through 5‐HT‐c‐Myc‐SLC6A4 axis, suggesting that SLC6A4 was an important molecule in tumorigenesis and metastasis.^28^ COMT, catechol‐O‐methyltransferase, a representative of catecholamine neurotransmitter pain genes. It has been reported that COMT gene polymorphism was related to the prognosis of KIRC by counteracting the catechol‐estrogen effect.^29^ In prostate cancer, COMT expression can inhibit the migration ability of tumor cells and increase the occurrence of cell apoptosis.^30^ In our study, high expression levels of SLC6A4 and COMT were associated with poor prognosis in KIRC. Therefore, they may be potential biomarkers and genes of interest for KIRC treatment.

### 4.3 Opioid receptor pain genes in KIRC

OPRD1, opioid receptor, delta 1, a representative of opioid receptor pain genes. The correlation between opioid receptor and tumor prognosis was controversial. TLR4, OPRL1, and OGFR opioid receptor genes have been reported to be associated with renal cancer progression.^31^, ^32^ An intriguing research result showed that was that mu, delta, and kappa opioid receptor genes had low expression in normal renal tissue and KIRC, but substantial genetic variability was observed in tumor cells.^33^ This meant that opioid receptor genes may play an essential role in KIRC through other mechanisms. Previous retrospective studies have suggested that the use of opioids may promote tumor progression, thereby negatively affecting the survival of patients with advanced cancer.^34^ There was also evidence that opioids use was associated with indirect immunosuppression, possibly through suppression of natural killer cell activity or interference with immune responses, leading to tumor invasion.^35^ Our study firstly found that OPRD1 expression was significantly upregulated in KIRC and correlated with the sensitivity of anti‐PD‐1 therapy.

### 4.4 Drug metabolism enzyme pain genes in KIRC

CYP2D6, cytochrome P450, family 2, subfamily D, polypeptide 6, a representative of cytochrome P4502D6 enzyme pain genes. Decreased CYP2D6 activity or the use of CYP2D6 inhibitors may reduce the clinical efficacy of tamoxifen and lead to an increased recurrence rate of breast cancer.^36^ CYP3A4, cytochrome P450, family 3, subfamily A, polypeptide 4, a representative of cytochrome P4503A4 enzyme pain genes. CYP3A4 was responsible for the metabolism of a variety of anticancer drugs, which may lead to an increased risk of side effects or toxicity or a decreased efficacy when mutated in the gene.^37^ cytochrome P450 enzymes can mediate the production of arachidonic acids, which was an important signaling mediator for inflammation, pathological angiogenesis, and destruction of vascular stability, eventually causing tumor growth and invasion.^38^ Of note, our study also showed that the drug metabolism enzyme genes, especially CYP2D6 and CYP3A4 genes, were associated with sensitivity of anti‐PD‐1 and PD‐L1 therapy.

## 5 CONCLUSION

Our study uncovered a mechanism for the effect of pain genes on KIRC outcome via the modulation of associated co‐expression gene networks, gene variation, and tumor Immunotherapy. SP1, SLC6A4, COMT, OPRD1, CYP2D6, and CYP3A4 were the new potential biomarkers for prognosis and treatment in KIRC.

## AUTHOR CONTRIBUTIONS

**Xiao‐Yu Zhuang**: Investigation; writing—original draft; writing—review and editing. **Ming Li**: Project administration; writing—original draft; writing—review and editing. **Da‐Ming Xu**: Investigation; methodology; writing—original draft. **Shu‐Bin Lin**: Investigation; methodology. **Zheng‐Liang Yang**: Resources; supervision. **Teng‐Yu Xu**: Supervision; visualization. **Jun Yin**: Conceptualization; project administration.

## CONFLICT OF INTEREST STATEMENT

The authors declare no conflict of interest.

## ETHICS STATEMENT

The study protocols were conducted according to the principles of the Declaration of Helsinki and were approved by the Scientific and Medical Ethical Committee of the Second Affiliated Hospital of Shantou University Medical College.

## TRANSPARENCY STATEMENT

The lead author Jun Yin affirms that this manuscript is an honest, accurate, and transparent account of the study being reported; that no important aspects of the study have been omitted; and that any discrepancies from the study as planned (and, if relevant, registered) have been explained.

## Data Availability

The data used to support the findings of this study are available from the corresponding author upon request.
